# Spillover Effects of Medicare Advantage on Traditional Medicare Beneficiaries With Prostate Cancer

**DOI:** 10.1002/cam4.70796

**Published:** 2025-03-20

**Authors:** Arnav Srivastava, Samuel R. Kaufman, Xiu Liu, Avinash Maganty, Addison Shay, Mary Oerline, Christopher Dall, Kassem S. Faraj, Paula Guro, Dawson Hill, Thuy Nguyen, Lindsey A. Herrel, Brent K. Hollenbeck, Vahakn B. Shahinian

**Affiliations:** ^1^ Dow Division of Health Services Research, Department of Urology University of Michigan Ann Arbor Michigan USA; ^2^ Department of Urology Massachusetts General Hospital Boston Massachusetts USA; ^3^ Department of Health Management and Policy University of Michigan School of Public Health Ann Arbor Michigan USA

**Keywords:** active surveillance, financial incentives, Medicare, medicare advantage, prostate cancer

## Abstract

**Introduction:**

Medicare Advantage (MA) managed care plans, now chosen by 51% of Medicare beneficiaries, are incentivized to constrain healthcare spending and utilization, a shift in financial incentives compared to Traditional Medicare's fee‐for‐service payment model. Beyond its primary beneficiaries, MA's mechanisms to constrain utilization may impact Traditional Medicare beneficiaries with prostate cancer through “spillover” effects on physician behavior.

**Methods:**

From a 20% sample of Medicare claims, we identified patients diagnosed with prostate cancer from 2016 to 2019. We calculated MA penetration [MA beneficiaries/(Traditional Medicare and MA beneficiaries)] at the practice‐level. We assessed the relationship between practice‐level MA penetration and two measures of quality—potential overtreatment (i.e., treatment among those with > 75% noncancer mortality within 10 years of diagnosis) and confirmatory testing (repeat prostate biopsy, MRI, or genomic test)—using a multilevel logistic regression. We also assessed two measures of utilization, price standardized spending (i.e., global utilization) and overall treatment.

**Results:**

We identified 41,092 patients. Median practice‐level MA penetration was 33% (IQR 23%–43%). Increasing practice‐level MA penetration was associated with increased odds of overall treatment among all Traditional Medicare beneficiaries (adjusted OR 1.03 (95% CI 1.01–1.05), *p* = 0.01, per 10% increase in MA penetration). However, MA penetration was not associated with our quality measures, potential overtreatment and confirmatory testing, or price‐standardized spending.

**Conclusions:**

MA penetration at the urology practice‐level varies considerably. In men with prostate cancer, greater practice‐level MA penetration was associated with increased odds of treatment, but not overall utilization—even where it might influence quality.

## Graphical abstract

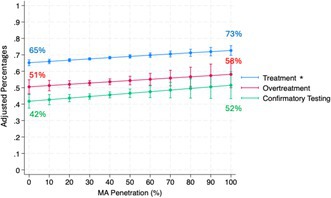

## 1 Background

Physician discretion often contributes substantially to the variation in the use and implementation of conservative management in prostate cancer [1, 2]. Nearly 40% of men with low‐risk disease undergo immediate treatment, despite being unlikely to benefit [3]. Even in men initiating active surveillance, which forgoes immediate treatment in favor of periodic reassessment of cancer risk, most do not receive all guideline‐recommended testing [4, 5, 6, 7]. Importantly, up to 60% of men miss guideline‐recommended confirmatory testing in the first year of active surveillance [8, 9], potentially underestimating the true biological nature and extent of the disease.

Given the importance of physician discretion, prostate cancer care is sensitive to incentives embedded in the delivery system, explaining some of the variation in clinical practice [10]. For example, the fee‐for‐service model of Traditional Medicare may promote utilization and treatment in older, sicker men, representing potential overtreatment [11, 12]. However, volume‐based incentives traditionally associated with Medicare payment policy have shifted substantially over the past two decades due to the rise of Medicare Advantage (MA) enrollment. MA managed care plans, now covering 51% of Medicare's 60 million beneficiaries, operate in a capitated payment system that emphasizes cost constraint and reduction of low‐value healthcare [13]. MA constrains cost and utilization through mechanisms that directly influence physician behavior, such as narrow networking and prior authorization requirements [14, 15, 16]. Beyond MA's primary beneficiaries, increased MA penetration may influence care among men covered by Traditional Medicare through spillover effects. As proposed by the “norms hypothesis,” a physician's practice style is often a function of the average health insurance of their patients, and they apply it similarly across their patient panel without regard to an individual's insurance [17]. Under this premise, a physician who predominantly sees patients covered by managed care plans will be biased towards less utilization (e.g., less testing or treatment), even when treating their fraction of patients under fee‐for‐service plans. In clinical contexts such as post‐acute care use after stroke, spillover effects of MA on the Traditional Medicare population suggest constraints on healthcare utilization [18, 19]. These constraining influences may not only shift treatment and testing patterns but also may have mixed effects on quality in prostate cancer.

We hypothesized that men managed by urology practices facing higher MA penetration would be associated with lower odds of potential overtreatment in men with a high risk of noncancer mortality, improving quality. However, these practices would also exhibit lower odds of confirmatory testing in men initiating active surveillance, lowering quality. Further, we hypothesized that higher practice‐level MA penetration would be associated with lower price‐standardized spending (i.e., global utilization) and overall treatment.

## 2 Methods

### 2.1 Study Population

From a 20% national sample of Medicare claims, we identified men with newly diagnosed prostate cancer between 2016 and 2019, using a validated algorithm with 99.8% specificity and 88.7% positive predictive value [20]. In assessing spillover effects of MA penetration, our analysis included only men covered by Traditional Medicare, and outcomes of those enrolled in MA plans were not assessed. Men included in our analysis had a minimum of 12 months of follow‐up and data available through December 31, 2020. We included only those with continuous enrollment in Medicare Parts A and B in the year before and after their prostate cancer diagnosis.

### 2.2 Medicare Advantage Practice Penetration

An empirical measure of MA penetration (i.e., proportion of Medicare beneficiaries covered by MA) was derived at the urology practice‐level and served as the study's primary exposure. First, as described in previous work, we defined urology practice markets (i.e., the geographical boundaries from which a practice draws its patients), using the variable market approach [21, 22, 23, 24]. Briefly, we established each practice's market as the minimum radius between zip code centroids of the practice and the patient that captured at least 75% of all Medicare payments to the practice each year. Notably, the practice market was defined by all patients seen in a given year, independent of diagnosis and gender. For practices with markets spanning multiple zip codes, we weighted each zip code based on its contribution of payments to a practice's market.

Using the Medicare Beneficiary Summary File, we then estimated the proportion of patients with MA [MA beneficiaries / (MA + Traditional Medicare beneficiaries)] at the zip code level. We then applied this proportion to each urology practice market, based on the weighted contribution of zip codes previously described. For example, in a practice market spanning three zip codes, Zip Codes A (MA penetration = 20%), B (MA penetration = 50%), and C (MA penetration = 40%), we would calculate their contribution to the practice—60%, 30%, 10%, respectively. Taking the weighted average of its zip codes [$\text{Zipcode}\;A\;\left(0.20\times 0.60\right)+\text{Zipcode}\;B\;\left(0.50\times 0.30\right)+\text{Zipcode}\;C\;\left(0.40\times 0.10\right)=0.31$], allowed us to derive the urology practice‐level MA penetration.

After determining urology practice‐level MA penetration, Traditional Medicare beneficiaries were assigned to practices as per previous methods [25, 26]. Briefly, all urologists providing care to a patient within the first year of a prostate cancer diagnosis were identified using their National Provider Identifier number. If a patient saw more than one urologist in the first year after their prostate cancer diagnosis, the patient was assigned to the urologist having 75% or more of the associated visits. Then urologists were assigned to practices in each year using tax‐identification numbers. With links between patients and urologists as well as between urologists and practices, we were able to assign patients to urology practices.

### 2.3 Primary Outcomes—Quality

Our study assessed two primary outcomes in prostate cancer quality, both measured at the patient level. First, we measured potential overtreatment. Given prostate cancer's protracted natural history, clinical guidelines recommend against treatment in older, sicker patients with a high risk of noncancer mortality [5, 6]. Using a previously validated model with high discrimination (C statistic = 0.82), we estimated noncancer mortality risk and identified a subgroup of men unlikely to benefit from treatment (i.e., noncancer mortality risk > 75% within 10 years of diagnosis) [27].

The second quality measure was the use of confirmatory testing in men initiating active surveillance. We identified men undergoing active surveillance using a previously validated algorithm with 97% specificity and 92% negative predictive value [28]. The men identified in the algorithm are a healthier subset of patients (< 75 years old with Charlson comorbidity index < 3). We considered men to have undergone confirmatory testing if they received at least one magnetic resonance imaging study, prostate biopsy, or genomic test within 12 months of diagnosis. Receiving a confirmatory test within 12 months of diagnosis is recommended by clinical guidelines, as up to 40% of men on active surveillance harbor more aggressive or extensive disease not found on their initial diagnostic biopsy [6, 10]. Confirmatory testing is particularly important in this healthier subset of men who have a greater lifetime likelihood of cancer progression and may eventually require treatment. Incentives afforded by increasing MA penetration to reduce spending may lead to underuse of confirmatory testing in men initiating surveillance.

### 2.4 Secondary Outcomes—Utilization

As a secondary outcome, we assessed price standardized spending, a global measure of healthcare utilization [22]. We summed price standardized spending, inflation adjusted to 2019 dollars, for Medicare claims associated with a prostate cancer diagnosis code during the 12‐month period after diagnosis. We hypothesized that higher practice‐level MA penetration would be associated with lower price standardized spending. Finally, we measured the odds of prostate cancer treatment, defined as either radiation therapy or surgery (i.e., radical prostatectomy) in all men regardless of noncancer mortality risk. Incentives to constrain spending in a practice market highly penetrated by MA may reduce treatment and encourage the adoption of more conservative management strategies. Given the potential for these constraining spillover effects, we hypothesized that Traditional Medicare beneficiaries managed by practices with higher MA penetration would have lower odds of undergoing treatment.

Though not statistically significant, we found that practices with higher MA penetration typically had lower price‐standardized spending. However, MA penetration was associated with increased odds of treatment (radiation therapy or surgery). Given that the association with MA penetration pointed in opposite directions for our two measures of utilization, we performed a post hoc analysis among men receiving treatment for their prostate cancer. As prior work demonstrates higher Medicare spending among patients treated with radiation therapy [29], we hypothesized that increased MA penetration would be associated with lower use of radiation therapy among patients undergoing treatment—and thereby higher use of surgery.

### 2.5 Statistical Analysis

We examined clinical characteristics of men by the MA penetration of their managing practice. To assess the relationship between practice‐level MA penetration and our four outcomes, we fit a multilevel model for each outcome. The multilevel model allowed us to account for the nested nature of our data (i.e., patients within practices). For our dichotomous outcomes—treatment, potential overtreatment, and confirmatory testing—we used logistic regression models. We fit a negative binomial model for the price standardized spending outcome. In all cases, we modeled MA penetration as a continuous variable (i.e., per 10% increase in MA penetration in a urology practice market). From the adjusted models, adjusted probabilities of dichotomous outcomes and spending were estimated using postestimation commands (i.e., the margins command in STATA). Regression models were adjusted for age, ethnicity, socioeconomic status, Charlson Comorbidity Index, rurality, year of diagnosis, United States Region, and practice type. We characterized practice type as small urology practice (1–2 urologists), medium urology practice (3–9 urologists), large urology practice (10 or more urologists), subspecialty group (only specialty physicians, not limited to urologists), multispecialty group (physicians from multiple specialties, including primary care), and hospital integrated.

Analyses were conducted using STATA version 18 software (College Station, TX). All statistical tests used *p* < 0.05 to define statistical significance. Our institutional review board deemed this study exempt from regulation.

## 3 Results

### 3.1 Cohort Characteristic and Practice‐Level MA Penetration

We identified 41,092 patients meeting inclusion criteria. Median practice‐level MA penetration across 6784 practice‐years was 33% (IQR 23%–43%) (Figure S1). Table 1 illustrates cohort characteristics, grouping men by the MA penetration quartile of their managing practice. Age and Charlson Comorbidity Index were well balanced between the quartiles. However, patients managed by practices highly penetrated by MA were more likely to live in cities (54% in Quartile 4 vs. 39% in Quartile 1) and the West region of the United States (31% in Quartile 4 vs. 11% in Quartile 1). They were also more commonly diagnosed with prostate cancer in later calendar years (diagnosed in 2019: 35% in Quartile 4 vs. 19% in Quartile 1).

**TABLE 1:** Cohort demographics by Medicare Advantage penetration quartile.

Variable		Quartile 1	Quartile 2	Quartile 3	Quartile 4
MA Penetration %	Mean, SD	17% (6%)	29% (3%)	39% (3%)	52% (7%)
Age (year)	Mean, SD	73 (5)	73 (5)	73 (5)	73 (5)
Race	White (%)	9060 (82%)	9671 (86%)	9821 (85%)	5872 (82%)
	Black (%)	1247 (11%)	850 (8%)	868 (8%)	545 (8%)
	Other (%)	771 (7%)	754 (7%)	853 (7%)	780 (11%)
CCI	0 (%)	6248 (56%)	6284 (56%)	6465 (56%)	3958 (55%)
	1 (%)	2126 (19%)	2028 (18%)	2075 (18%)	1256 (18%)
	2 (%)	1443 (13%)	1558 (14%)	1586 (14%)	975 (14%)
	3 or greater (%)	1261 (11%)	1405 (13%)	1416 (12%)	1008 (14%)
SES Tertile	1 (%)	3560 (32%)	3310 (29%)	3168 (27%)	1971 (27%)
	2 (%)	3845 (35%)	3857 (34%)	4107 (36%)	2694 (37%)
	3 (%)	3673 (33%)	4108 (36%)	4267 (37%)	2532 (35%)
Urban–Rural Scale	City (%)	4286 (39%)	5136 (46%)	6025 (52%)	3816 (54%)
	Metro county (%)	3629 (33%)	3903 (35%)	3734 (32%)	2325 (33%)
	Near metro area (%)	2661 (24%)	1921 (17%)	1567 (14%)	910 (13%)
	Rural (%)	439 (4%)	291 (3%)	200 (2%)	78 (1%)
Diagnosis year	2016 (%)	3258 (29%)	2560 (23%)	2452 (23%)	1227 (17%)
	2017 (%)	2992 (27%)	2860 (25%)	2657 (23%)	1542 (21%)
	2018 (%)	2716 (25%)	2970 (26%)	3062 (26%)	1939 (27%)
	2019 (%)	2112 (19%)	2885 (26%)	3371 (26%)	2489 (35%)
USA region	Northeast (%)	3252 (30%)	3013 (27%)	1458 (13%)	1031 (15%)
	Southeast (%)	3756 (34%)	2918 (26%)	3321 (29%)	1691 (24%)
	Midwest (%)	2233 (20%)	2688 (24%)	2839 (25%)	1666 (23%)
	Southwest (%)	562 (5%)	1589 (14%)	1729 (15%)	551 (8%)
	West (%)	1212 (11%)	1043 (9%)	2179 (19%)	2190 (31%)
Practice type	Small urology practice	1654 (15%)	1241 (11%)	988 (9%)	1055 (15%)
	Medium urology practice	2617 (24%)	1652 (15%)	1731 (15%)	1371 (19%)
	Large urology practice	2764 (25%)	3122 (28%)	3081 (26%)	1579 (22%)
	Subspecialty group	306 (3%)	209 (2%)	398 (3%)	120 (2%)
	Multispecialty group	3201 (29%)	4314 (38%)	4429 (38%)	2433 (34%)
	Hospital integrated practice	536 (5%)	737 (7%)	915 (8%)	639 (9%)

### 3.2 Primary Outcomes—Quality

**FIGURE 1:**
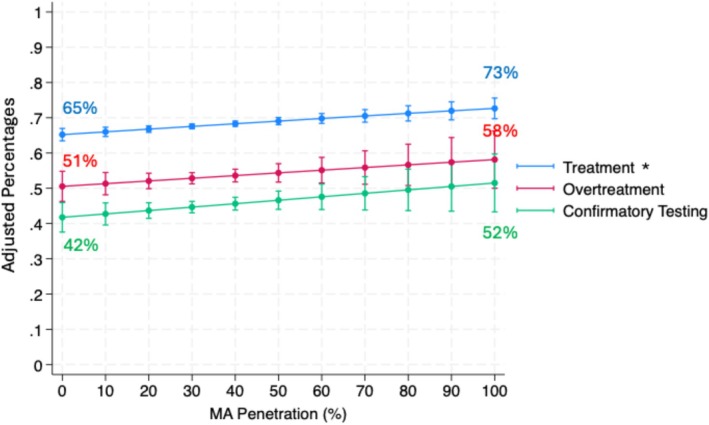
Adjusted probabilities of binary outcomes by medicare advantage penetration. *Indicates statistical significance. Models adjusted for age, ethnicity, socioeconomic status, Charlson Comorbidity Index, rurality, year of diagnosis, USA region, and practice type.

After adjusting for patient characteristics, MA penetration was not associated with potential overtreatment (adjusted OR 1.04 (95% CI 0.98, 1.10), *p* = 0.22, per 10% increase in MA penetration) in those with competing health risks (> 75% noncancer mortality within 10 years of diagnosis). This represents an increase of 0.8% (95% CI −0.4%, 2.0%) in the adjusted probability of potential overtreatment per 10% increase in MA penetration. Among men initiating active surveillance, the adjusted probability of receiving a confirmatory test increased by 1.2% (95% CI 0.0%, 2.3%) for each 10% increase in MA penetration but did not reach statistical significance (adjusted OR 1.04 (95% CI 0.99, 1.10) *p* = 0.11; Figure 1, Table S1).

### 3.3 Secondary Outcomes—Utilization

**FIGURE 2:**
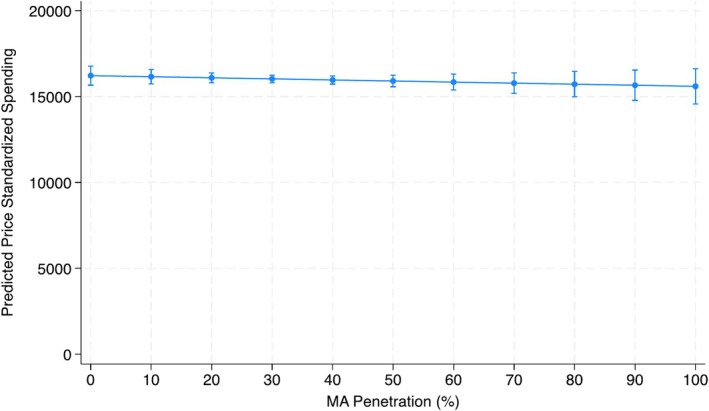
Adjusted price standardized spending by medicare advantage penetration. Model adjusted for age, ethnicity, socioeconomic status, Charlson Comorbidity Index, rurality, year of diagnosis, USA region, and practice type.

After adjusting for patient characteristics, practice‐level MA penetration was not associated with global utilization, as measured by price standardized spending (Incident Rate Ratio 1.00 95% CI (0.99, 1.01), *p* = 0.49; Table S1). This represents a $54.68 decrease in adjusted price standardized spending per 10% increase in MA penetration (Figure 2), though not statistically significant (95% CI: −$208.82, $99.47). MA penetration was associated with an increase in treatment among all patients (adjusted OR 1.04 (95% CI 1.01, 1.06), *p* = < 0.001, per 10% increase in MA penetration; Table S1). For each 10% increase in MA penetration, 0.7% (95% CI 0.3%, 1.2%) more patients in a practice would undergo treatment (Figure 1).

**FIGURE 3:**
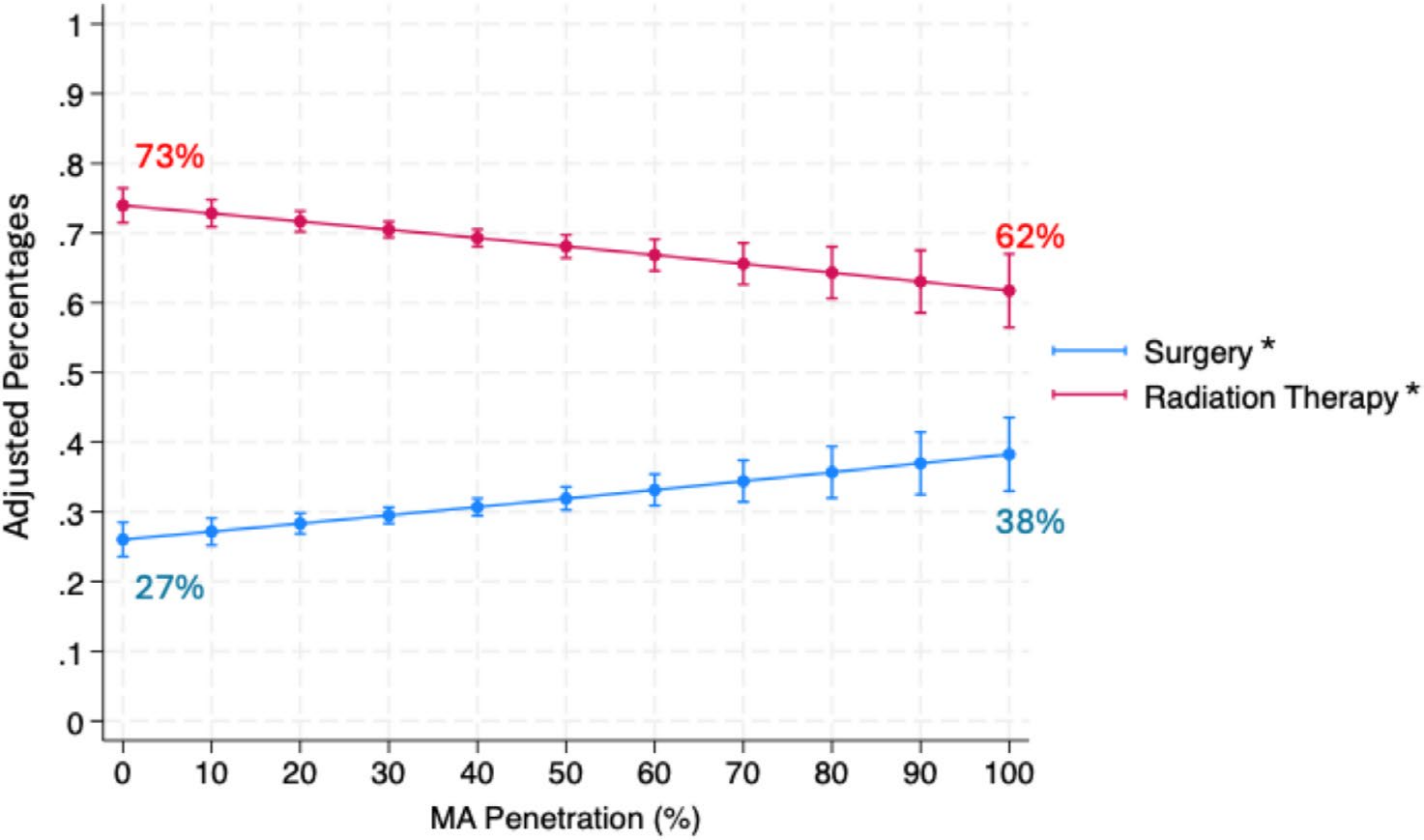
Adjusted Probability of Treatment Modality by Medicare Advantage Penetration Among Patients Receiving Treatment. *Indicates statistical significance. Models adjusted for age, ethnicity, socioeconomic status, Charlson Comorbidity Index, rurality, year of diagnosis, USA region, and practice type.

Lastly, we modeled the odds of treatment modality, radiation therapy or surgery, among men receiving prostate cancer treatment. Increasing practice‐level MA penetration was associated with a decrease in radiation therapy (adjusted OR 0.93 (95% CI 0.89, 0.97), *p* < 0.001; Table S1). Among men receiving treatment, for every 10% increase in MA penetration, the adjusted probability of undergoing radiation therapy decreased by 1.1% (95% CI −1.8%, −0.4%). Reciprocally, for every 10% increase in MA penetration, the adjusted probability of undergoing surgery increased by 1.1% (95% CI 0.4%, 1.8%; Figure 3).

## 4 Discussion

In this study, we examined the spillover effects of urology practice‐level MA penetration on prostate cancer care among men covered by Traditional Medicare. The median practice‐level MA penetration was 33% (i.e., 33% of Medicare beneficiaries in practice's market enrolled in MA plans), which varied substantially (IQR 23%–43%). Our analyses suggest that greater practice‐level MA penetration was associated with increased treatment among Traditional Medicare beneficiaries, but without changes in quality (i.e., potential overtreatment and confirmatory testing), challenging our original hypothesis.

Our original hypothesis draws from prior work, which suggests that MA's incentives to limit spending might broadly influence physician behavior and decrease utilization among Traditional Medicare beneficiaries through constraining spillover effects (e.g., a provider facing high MA penetration grows to expect significant prior authorization requirements and subsequently decreases their use of treatment) [20]. Further, the extent of these spillover effects would be magnified in practices more penetrated by MA. In the context of prostate cancer, recent literature has characterized the burdens of prior authorization among radiation oncology practices [30]. Additionally, publicly available resources, such as Humana's prior authorization search tool, demonstrate that for most MA plans, radiation therapy and radical prostatectomy require prior authorization [31]. Supporting this mechanism of spillover effects in another clinical scenario, a recent study demonstrated that per 10% increase in MA market‐level penetration, Traditional Medicare beneficiaries had a 1.14% and 0.82% relative decrease in post‐acute care following hospitalizations for congestive heart failure and stroke, respectively [20].

Our observation of a positive relationship between urology practice‐level MA penetration and treatment among Traditional Medicare beneficiaries is supported by the mixed‐economy model. The model proposes that, when faced with reimbursement that varies by payer, providers may preferentially offer services first in settings where reimbursement is more attainable (i.e., Traditional Medicare) or higher [32, 33, 34]. While payments between MA and Traditional Medicare are similar [35], reimbursement for treating Traditional Medicare beneficiaries may be more readily attainable as physicians do not need to overcome administrative barriers (e.g., prior authorizations). Second, to counteract these barriers, practices may require substantial investment and time, which decrease their profit margins in these managed care settings. Clinical contexts primarily under the urologist's discretion—recommending radiation therapy (where radiation oncology referrals must consider in‐network providers) or testing during active surveillance—may be particularly influenced by these incentives. These features of MA plans which restrict spending among their primary beneficiaries may further shift utilization towards the Traditional Medicare population, in which such barriers do not exist.

Our study has several limitations. First, our analysis is cross‐sectional in nature, as the exposure is MA penetration in a practice‐year. As a result, our analysis may not adequately address secular trends in prostate cancer within a practice (i.e., increasing use of conservative management over time) or changes in practice‐level MA penetration over time. Second, practices may respond differently to increased MA penetration, depending on their existing practice patterns and MA penetration at the start of the study period. Acknowledging this, we used a multilevel model to account for inter‐practice variation and incorporated fixed effects for each year in the study. Third, to measure a urology practice's MA penetration, we leveraged the variable market approach and used a weighted average of zip code‐level MA penetration. However, within these markets, differences in patient selection or practice reputation may lead to nuances in a practice's patient panel not captured by the variable market approach, particularly when two or more practices have overlapping markets. Lastly, as a limitation of Medicare claims data, we cannot capture cancer risk or stage, which are important determinants of the appropriateness of treatment. However, as detailed by clinical guidelines, conservative management is the preferred strategy in older, sicker men with prostate cancer. These men are at the greatest risk of overtreatment, as they are unlikely to benefit from treatment even when facing high‐risk tumors [5, 6]. Additionally, in prior analyses, we have demonstrated that cancer risk characteristics are well balanced across practices, mitigating the possibility that practice patterns are driven by differences in patient cancer risk [36].

Our results have several policy‐related implications. First, in the context of surgical subspecialty care, our results offer a counterbalance to prior work which suggests that increasing MA penetration would exert a constraining effect on utilization among patients insured by Traditional Medicare. MA was originally developed in the hopes that private competition and managed care plans would provide lower cost, more efficient care over the entire Medicare population [37, 38]. However, concerns of upcoding of patient risk in MA plans [39], favorable selection [40], and overpayments [41] in markets heavily penetrated by MA have called into question whether MA truly improves efficiency in the Medicare program. Our work pairs with these concerns, in that MA penetration may not constrain utilization in the Traditional Medicare population for surgical subspecialty care.

Second, to mitigate spillover effects of alternative payment models and their unpredictable effects on quality, policymakers may increasingly consider the implementation of all‐payer models. For example, the Maryland All‐Payer Model blends payments from government and private payers and provides a global budget for hospitals to operate under. By equilibrating payments, the all‐payer model may reduce cost‐shifting and differences in care patterns along the lines of payment model incentives [42]. However, the capitated nature of all‐payer models may lead to lower utilization in contexts where healthcare is essential [43]. Further, in an effort to reduce hospital‐acquired conditions and readmissions, all‐payer models may incentivize preferential treatment of healthier patients, while restricting access to those with multimorbidity [44, 45].

## 5 Conclusions

MA penetration at the urology practice‐level varies considerably. Higher MA penetration was associated with increased treatment, namely surgery, among patients with Traditional Medicare without changes in quality. Our work suggests that, in the context of prostate cancer, increasing MA penetration may not always be associated with constrained utilization among Traditional Medicare beneficiaries.

## Author Contributions

**Arnav Srivastava:** conceptualization (equal), formal analysis (equal), investigation (equal), writing – original draft (equal), writing – review and editing (equal). **Samuel R. Kaufman:** formal analysis (equal), methodology (equal). **Xiu Liu:** data curation (equal), formal analysis (equal). **Avinash Maganty:** methodology (equal), writing – review and editing (equal). **Addison Shay:** data curation (equal), formal analysis (equal), methodology (equal). **Mary Oerline:** data curation (equal), formal analysis (equal). **Christopher Dall:** writing – review and editing (equal). **Kassem S. Faraj:** methodology (equal), writing – review and editing (equal). **Paula Guro:** project administration (equal). **Dawson Hill:** project administration (equal), writing – review and editing (equal). **Thuy Nguyen:** conceptualization (equal), writing – review and editing (equal). **Lindsey A. Herrel:** supervision (equal), writing – review and editing (equal). **Brent K. Hollenbeck:** funding acquisition (equal), methodology (equal), project administration (equal), resources (equal), supervision (equal), visualization (equal), writing – review and editing (equal). **Vahakn B. Shahinian:** investigation (equal), methodology (equal), project administration (equal), resources (equal), supervision (equal), writing – review and editing (equal).

## Conflicts of Interest

Arnav Srivastava reports grant funding from the National Cancer Institute. Brent Hollenbeck reports fees from Elsevier Publishing for professional activities and grant funding by the National Cancer Institute, American Cancer Society, National Institute on Aging, and the Agency for Healthcare Research and Quality. Vahakn Shahinian reports grant funding from the National Cancer Institute, American Cancer Society, and Agency for Healthcare Research and Quality. The other authors declare no conflicts of interest.

## Supporting information


Data S1.

## Data Availability

The data underlying this article were provided by the Centers for Medicare and Medicaid Services and cannot be shared by the authors of this manuscript under the data use agreement.
