# Supplementary material for: Spillover Effects of Medicare Advantage on Traditional Medicare Beneficiaries With Prostate Cancer
Source: Cancer Med. 2025 Mar 20;14(6):e70796. doi: 10.1002/cam4.70796 (PMC11924284; doi:10.1002/cam4.70796)
Supplement: Supplementary file 1 — Data S1. [file CAM4-14-e70796-s001.docx]

**Supplement**

**Supplemental Figure 1: Variation of MA Penetration by Practice-Year**

| **Supplemental Table 1: Adjusted Regression Outcomes*** | | | | |
| --- | --- | --- | --- | --- |
|  | | | | |
| *Overtreatment (among those with >75% noncancer mortality)* | | | | |
|  | **OR** | **95%CI** | | **p value** |
| MA Penetration (per 10% increase) | 1.04 | 0.98 | 1.10 | 0.22 |
|  |  |  |  |  |
| *Confirmatory Testing (among patients on active surveillance)* | | | | |
|  | **OR** | **95%CI** | | **p value** |
| MA Penetration (per 10% increase) | 1.04 | 0.99 | 1.10 | 0.11 |
|  |  |  |  |  |
| *Price Standardized Spending* | | | | |
|  | **IRR** | **95%CI** | | **p value** |
| MA Penetration (per 10% increase) | 1.00 | 0.99 | 1.01 | 0.49 |
|  |  |  |  |  |
| *Treatment* | | | | |
|  | **OR** | **95%CI** | | **p value** |
| MA Penetration (per 10% increase) | 1.04 | 1.01 | 1.06 | <0.001 |
|  |  |  |  |  |
| *Radiation Therapy (among patients receiving treatment)* | | | | |
|  | **OR** | **95%CI** | | **p value** |
| MA Penetration (per 10% increase) | 0.93 | 0.89 | 0.97 | <0.001 |
|  |  |  |  |  |
| *Adjusted for age, ethnicity, socioeconomic status, Charlson Comorbidity Index, rurality, year of diagnosis, USA region, and practice type | | | | |
| MA = Medicare Advantage, IRR = incidence rate ratio, OR = odds ratio, 95%CI = 95% Confidence Interval | | | | |
